# Characterization and treatment of gemcitabine‐ and cisplatin‐resistant bladder cancer cells with a pan‐RAS inhibitor

**DOI:** 10.1002/2211-5463.13616

**Published:** 2023-04-29

**Authors:** Hirofumi Yoshino, Seiya Yokoyama, Motoki Tamai, Shunsuke Okamura, Sayaka Iizasa, Takashi Sakaguchi, Yoichi Osako, Satoru Inoguchi, Ryosuke Matsushita, Yasutoshi Yamada, Masayuki Nakagawa, Shuichi Tatarano, Akihide Tanimoto, Hideki Enokida

**Affiliations:** ^1^ Department of Urology, Graduate School of Medical and Dental Sciences Kagoshima University Japan; ^2^ Department of Pathology, Graduate School of Medical and Dental Sciences Kagoshima University Japan

**Keywords:** bladder cancer, cisplatin resistance, gemcitabine resistance, pan‐RAS inhibitor, RAS

## Abstract

Combination chemotherapy with gemcitabine and cisplatin (GC) is recommended as the primary treatment for advanced bladder cancer (BC). However, the benefits of this approach are limited owing to the acquisition of drug resistance. Here, we found that gemcitabine‐resistant and cisplatin‐resistant BCs do not exhibit cross‐resistance, and that these BCs exhibit different mRNA patterns, as revealed using RNA sequence analysis. To overcome drug resistance, we used the newly developed pan‐RAS inhibitor Compound 3144. Compound 3144 inhibited cell viability through suppression of RAS‐dependent signaling in gemcitabine‐ and cisplatin‐resistant BCs. RNA sequencing revealed that several genes and pathways, particularly those related to the cell cycle, were significantly downregulated in Compound 3144‐treated BCs. These findings provide insights into potential therapeutic strategies for treating BC.

## Graphical abstract

We found that gemcitabine‐resistant (GR) and cisplatin‐resistant (CR) bladder cancer cells did not exhibit cross‐resistance, which was supported by the result that these cells exhibited different mRNA patterns through RNA sequence analysis. The newly developed pan‐RAS inhibitor Compound 3144 suppressed both GR and CR cells through suppression of RAS‐dependent signaling.

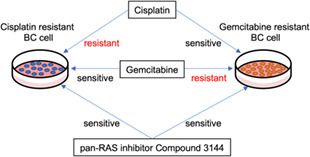

**Abbreviations**
ADC: antibody‐drug conjugate
BC: bladder cancer
CR: cisplatin‐resistant
CSC: cancer stem cell
dCK: deoxycytidine kinase
DMSO: dimethyl sulfoxide
ERK: extracellular signal‐regulated kinase
GC: gemcitabine and cisplatin
GR: gemcitabine‐resistant
IC_50_
: half‐maximal inhibitory concentration
MEME: minimum essential medium Eagle
PCoA: Principal coordinate analysis
PD‐1: programmed death receptor‐1
PD‐L1: programmed death ligand 1
RNAseq: RNA sequencing
RRM: ribonucleotide reductase catalytic subunit M
TCGA: The Cancer Genome Atlas

The prognosis of patients with advanced bladder cancer (BC) is poor because of frequent disease relapse and death, even if antitumor drug therapy and radiotherapy are temporarily successful in patients with recurrent or metastatic disease [1]. In particular, combination chemotherapy with gemcitabine and cisplatin (GC) is used as the first‐line antitumor therapy; however, its efficacy is limited [2, 3], and clinical trials of other antitumor therapies and various molecular‐targeted agents have failed to demonstrate efficacy [4, 5]. Recently, anti‐programmed death receptor‐1 (PD‐1) and programmed death ligand 1 (PD‐L1) antibodies have been clinically applied for the treatment of advanced or metastatic BC; however, the response rates to these drugs are insufficient, and therapeutic efficacy is limited [6, 7]. Thus, compared with other urological cancers, such as prostate cancer and renal cancer, there are fewer therapeutic options for patients with BC. Accordingly, further studies are needed to elucidate the mechanisms of acquisition of drug resistance and to develop novel therapeutic strategies.

We have previously generated several gemcitabine‐resistant BC cell lines (GR‐BOY, GR‐T24) [8] and cisplatin‐resistant (CR) BC cell lines (CR‐BOY, CR‐T24) [9]. Using gemcitabine‐resistant BC cells, we found that *microRNA‐99a‐5p* induces cellular senescence by targeting SWI/SNF‐related, matrix‐associated, actin‐dependent regulator of chromatin, subfamily D, member 1, a member of the SWI/SNF chromatin remodeling complex family [8]. Additionally, in cisplatin‐resistant BC cells, we reported that enoyl‐CoA hydratase and 3‐hydroxyacyl CoA dehydrogenase, which is involved in the degradation of long‐chain fatty acids in peroxisomes and in the β‐oxidation of fatty acids, contributes to cisplatin resistance through regulation of tumor‐suppressive *microRNA‐486‐5p* [9]. However, it is unclear whether there is cross‐resistance between GC and whether there are independent or common mechanisms of acquisition of drug resistance in BC cell lines.

HRAS, an isoform of RAS, was the first human oncogene found to be mutated in the BC cell line T24 in 1982 [10], and HRAS mutations are reported to be present in approximately 4% of BC cases [11]. Because KRAS and NRAS mutations also occur in many other cancers [12], there have been worldwide efforts to develop RAS inhibitors, including salirasib, which selectively disrupts the binding of the active RAS protein to the plasma membrane. Previously, we reported that salirasib requires high volumes to suppress BC cell lines in *in vitro* experiments and fails to suppress tumor growth *in vivo* [13]. The difficulty in developing RAS inhibitors is associated with the lack of binding sites on the RAS protein surface; however, new compounds that bind simultaneously to multiple sites on the activated and conformationally altered surface of the RAS protein have recently been developed [14]. The newly developed pan‐RAS inhibitor Compound 3144 suppresses RAS protein activity *in vitro* and *in vivo* and exhibits antitumor efficacy in cancer cells. However, it is unclear whether this novel inhibitor exerts antitumor effects in BC cells and in cells that have acquired drug resistance.

Accordingly, in this study, we first investigated whether there was cross‐resistance between gemcitabine‐ and cisplatin‐resistant BC cell lines. We then performed RNA sequencing (RNAseq) analysis to identify alterations in the cells that may support the presence or absence of cross‐resistance. Furthermore, as a novel therapeutic strategy against gemcitabine‐ and cisplatin‐resistant BC cells, we used the novel pan‐RAS inhibitor Compound 3144 in *in vitro* experiments. Finally, we investigated genes and pathways affected by Compound 3144 in gemcitabine‐ and cisplatin‐resistant BC cells using RNAseq analysis.

## Materials and methods

### BC cell lines and culture

We used 2 human BC cell lines (BOY and T24). BOY cells were established in our laboratory from a 66‐year‐old Asian male patient, who was diagnosed with stage IV BC with many lung metastases. T24 cells were obtained from American Type Culture Collection (Manassas, VA, USA). Using these cell lines, we established gemcitabine‐resistant BC cell lines (GR‐BOY and GR‐T24) and cisplatin‐resistant BC cell lines (CR‐BOY and CR‐T24), as previously reported [8, 9]. These cell lines were cultured in minimum essential medium Eagle (MEME) supplemented with 10% fetal bovine serum at 37 °C in a humidified, 5% CO_2_ incubator.

### Pan‐RAS inhibitor

For *in vitro* experiments, the pan‐RAS inhibitor Compound 3144 (CAS 1835283‐94‐7; ProbeChem, Inc., Shanghai, China), solubilized in dimethyl sulfoxide (DMSO), was used. The Compound 3144/DMSO solution was prepared in MEME at different concentrations, and the solutions were added to cell culture plates at final inhibitor concentrations of 1, 2, 3, 4, or 5 μm. The DMSO concentration was adjusted to 0.1%.

### Cell proliferation, migration, and invasion assays

To evaluate cell proliferation, we used XTT assays. BOY and T24 cells were seeded in 96‐well plates (1 × 10^3^ cells/well) with 100 μL medium per well. We determined the extent of cell proliferation 96 h after seeding using a Cell Proliferation Kit II (Roche Diagnostics GmbH, Mannheim, Germany). When using cisplatin, gemcitabine, or Compound 3144, we added 10 μL of the stock solution, adjusted to 10‐times the target concentration. Inhibition data were used to calculate the half‐maximal inhibitory concentration (IC_50_) values using nonlinear, four‐parameter, variable slope equation software (graphpad prism ver. 8.00 for Windows; GraphPad Software, San Diego, CA, USA). Wound healing assays were used to assess cell migration activity. Cells (2 × 10^5^ cells/well) were plated in 6‐well plates, and after 48 h of incubation, the cell monolayer was scraped using a P‐1000 micropipette tip. The initial gap length (0 h) and the residual gap length 24 h after wounding were calculated from photomicrographs. For cell invasion assays, we used modified Boyden chambers consisting of Matrigel‐coated Transwell membrane filter inserts with 8‐μm pores in 24‐well tissue culture plates (BD Biosciences, San Jose, CA, USA). The cells that passed through the pores and attached to the surface of the chamber were counted from photomicrographs.

### Three‐dimensional spheroid culture system

To evaluate spheroid formation, we introduced three‐dimensional culture system (Cell‐able™; Toyo Gosei Co. Ltd. Tokyo, Japan). According to the manufacturer's protocol, BC cells were seeded in 96‐well plates (5 × 10^3^ cells/well) with 100 μL medium per well with control (DMSO) or Compound 3144 (5 μm). 48 h after incubation, spheroid formations were evaluated, respectively.

### Western blotting

Cell lysates were separated on NuPAGE 4–12% bis‐tris gels (Invitrogen, Carlsbad, CA, USA) and transferred to polyvinylidene difluoride membranes. Immunoblotting was performed with the following reagents from Cell Signaling Technology (Danvers, MA, USA): anti‐AKT antibodies (1 : 2000; cat. no. 4691), anti‐phospho‐AKT antibodies (1 : 1000; cat. no. 4060), anti‐extracellular signal‐regulated kinase (ERK) antibodies (1 : 2000; cat. no. 4695), and anti‐phospho‐ERK antibodies (1 : 1000; cat. no. 4370). Anti‐β‐actin antibodies (1 : 5000; cat. no. bs‐0061R) were obtained from Bioss (Beijing, China). Specific complexes were visualized using an enhanced chemiluminescence detection system (GE Healthcare), as described previously [15].

### RNA sequencing

An Isogen kit (Nippon Gene, Tokyo, Japan) was used for extraction of total RNA following the manufacturer's protocol. Total RNA from each cell was subjected to RNAseq, which was performed by Eurofins Japan. mRNA profiles were generated by single‐read deep sequencing using an Illumina HiSeq 4000 instrument (Illumina, San Diego, CA, USA).

### Statistical analysis

Cluster analysis was performed in r (version 4.1.2) using Euclidean distances with the vegan package and Ward.D2 linkage with the complex heatmap package. Principal coordinate analysis (PCoA) was performed using the distance calculated with the altGower method. Data are presented as means ± standard deviations of at least three independent experiments. The relationships between two groups were analyzed using Mann–Whitney *U* tests. The relationships among three or more variables and numerical values were analyzed using Bonferroni‐adjusted Mann–Whitney *U* tests. All analyses were performed using Expert statview software, version 5.0 (SAS Institute, Inc., Cary, NC, USA). Results with *P* values less than 0.05 were considered statistically significant.

### Analysis of a BC cohort with The Cancer Genome Atlas

In order to evaluate the genetic alteration of RAS genes such as HRAS, KRAS, and NRAS, a The Cancer Genome Atlas (TCGA) cohort database of 476 patients with BLCA was used through cBioPortal (http://www.cbioportal.org/public‐portal/).

### Ethics and standards for conducting of human

Our study was approved by the Bioethics Committee of Kagoshima University; written prior informed consent and approval were given by the patient from which the human BC cell line BOY was established. The study methodologies, approved by the ethics committee of Kagoshima University as 21S009, conformed to the standards set by the Declaration of Helsinki.

## Results

### IC_50_ values of cisplatin and gemcitabine for parental and drug‐resistant cells

**Fig. 1:**
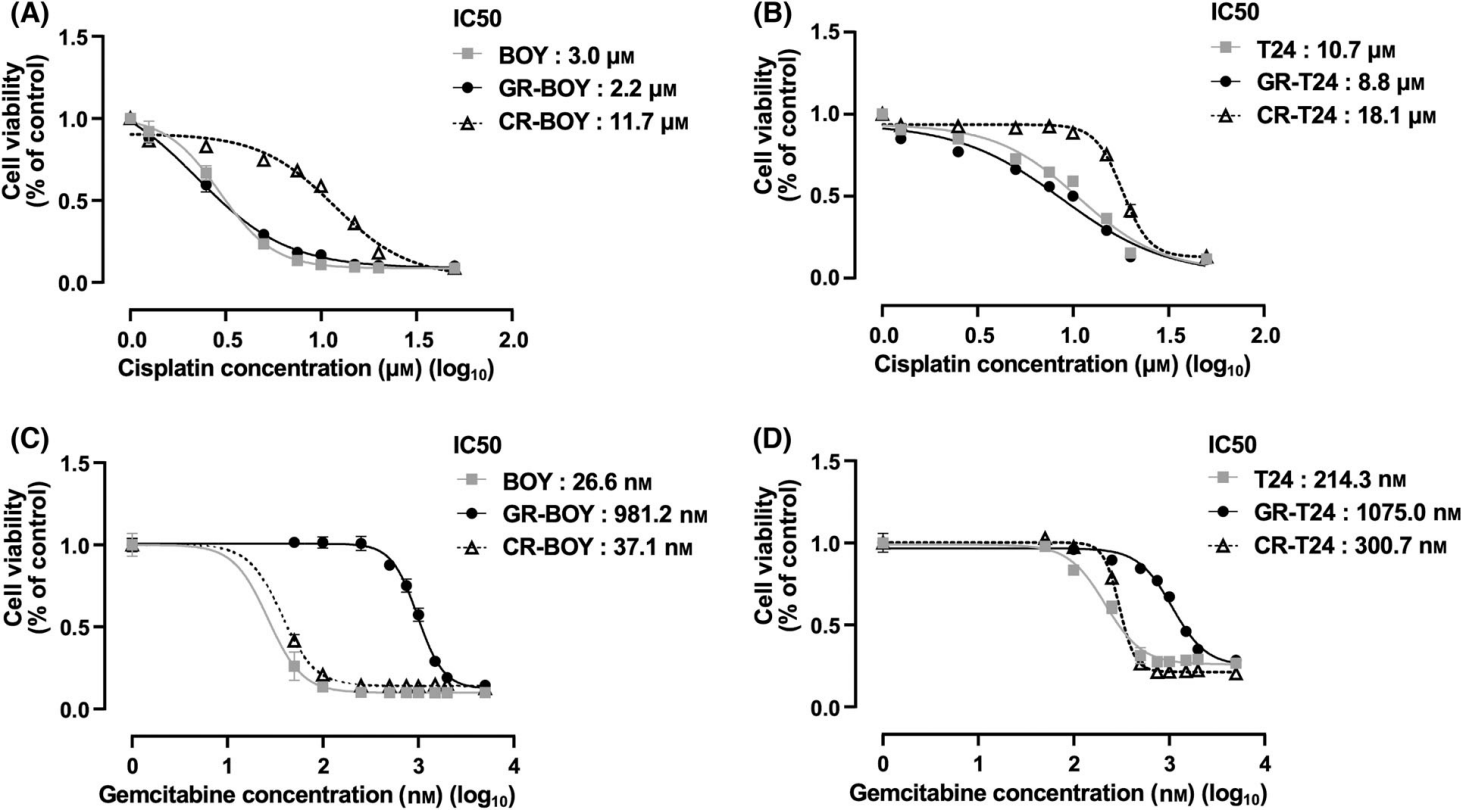
IC_50_ values of cisplatin and gemcitabine in parental and drug‐resistant cells, and RNAseq analyses of drug‐resistant cells. (A, B) The IC_50_ values of cisplatin in parental and drug‐resistant cells. (A) BOY/GEM‐R‐BOY/CDDP‐R‐BOY and (B) parental T24/GEM‐R‐T24/CDDP‐R‐T24. (C, D) The IC_50_ values of gemcitabine in parental and drug‐resistant cells (C) BOY/GEM‐R‐BOY/CDDP‐R‐BOY and (D) parental T24/GEM‐R‐T24/CDDP‐R‐T24. *n* = 6. Error bars indicate standard errors of the means.

To determine whether there was cross‐resistance of gemcitabine‐resistant cells to cisplatin and cisplatin‐resistant cells to gemcitabine, we calculated the IC_50_ values of both drugs in these cells. GR‐BOY and GR‐T24 did not show cisplatin resistance, similar to the parent cell lines, whereas CR‐BOY and CR‐T24 showed more than 5‐ and 2‐fold higher IC_50_ values compared with the GR cell lines (BOY IC_50_: 3.0 μm, GR‐BOY IC_50_: 2.2 μm, CR‐BOY IC_50_: 11.7 μm, Fig. 1A; T24 IC_50_: 10.7 μm, GR‐T24 IC_50_: 8.8 μm, CR‐T24 IC_50_: 18.1 μm, Fig. 1B). Similarly, CR‐BOY and CR‐T24 did not show gemcitabine resistance, similar to the parent cell lines, whereas GR‐BOY and GR‐T24 showed more than 26‐ and 3‐fold higher IC_50_ values compared with the CR cell lines (BOY IC_50_: 26.6 nm, GR‐BOY IC_50_: 981.2 nm, CR‐BOY IC_50_: 37.1 nm, Fig. 1C; T24 IC_50_: 214.3 nm, GR‐T24 IC_50_: 1075.0 nm, CR‐T24 IC_50_: 300.7 nm, Fig. 1D).

### RNAseq of parental and drug‐resistant cells

**Fig. 2:**
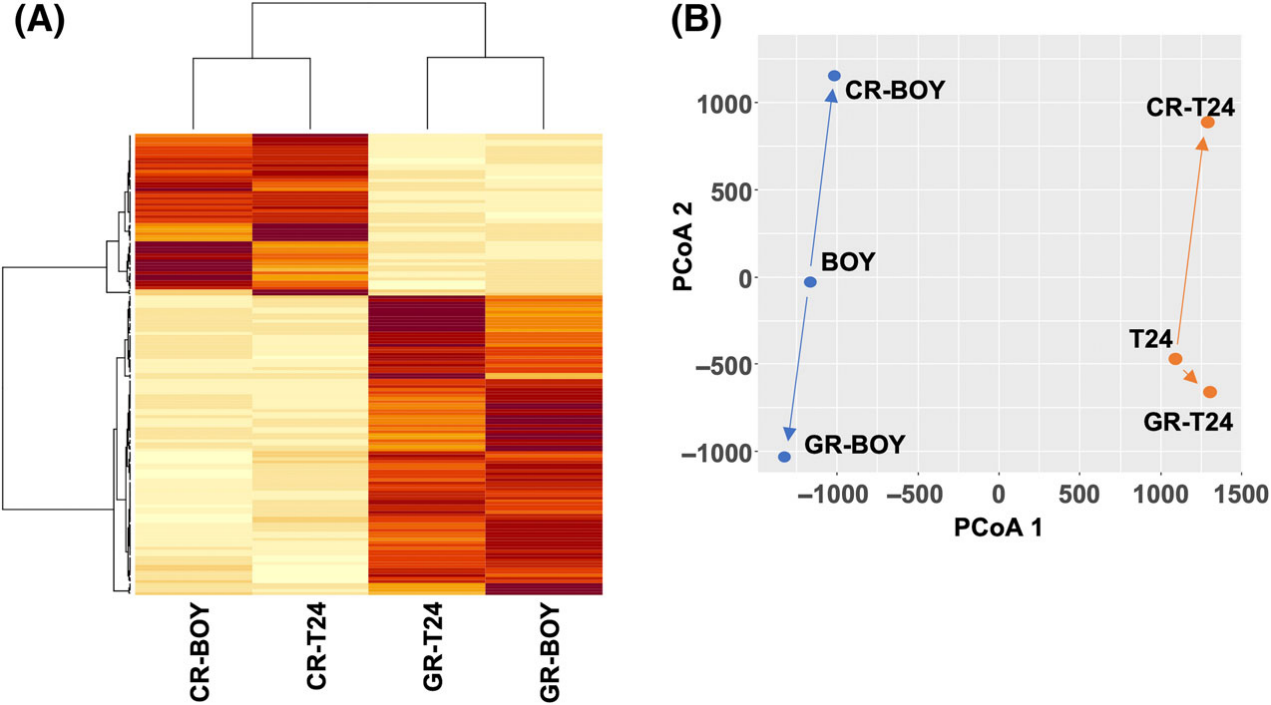
RNAseq analyses of drug‐resistant cells. (A) Cluster analysis of a select 402 gene dataset (2‐ or 0.5‐fold differences in expression) for identification of distinct clusters between gemcitabine resistance and cisplatin resistance based on parental cell lines. High mRNA expression levels are indicated in red, and low levels are indicated in yellow. GR: gemcitabine resistance, CR: cisplatin resistance. (B) PCoA of the samples based on the cell resistance type. The Mardia fit measure 1 was 0.606, and the Mardia fit measure 2 was 0.801 on PCoA using mRNA expression analysis.

Cluster analysis of mRNA expression levels did not show distinct profiles for drug‐resistant cell lines (Fig. S1). However, cluster analysis using a select 402 gene dataset (2‐ or 0.5‐fold differences in expression), demonstrated distinct clusters associated with gemcitabine resistance and cisplatin resistance (Fig. 2A). PCoA showed clear differences on the *y*‐axis between gemcitabine resistance and cisplatin resistance (Fig. 2B).

### Compound 3144 inhibited cell viability by suppressing RAS‐dependent signaling in gemcitabine‐resistant BC cells

**Fig. 3:**
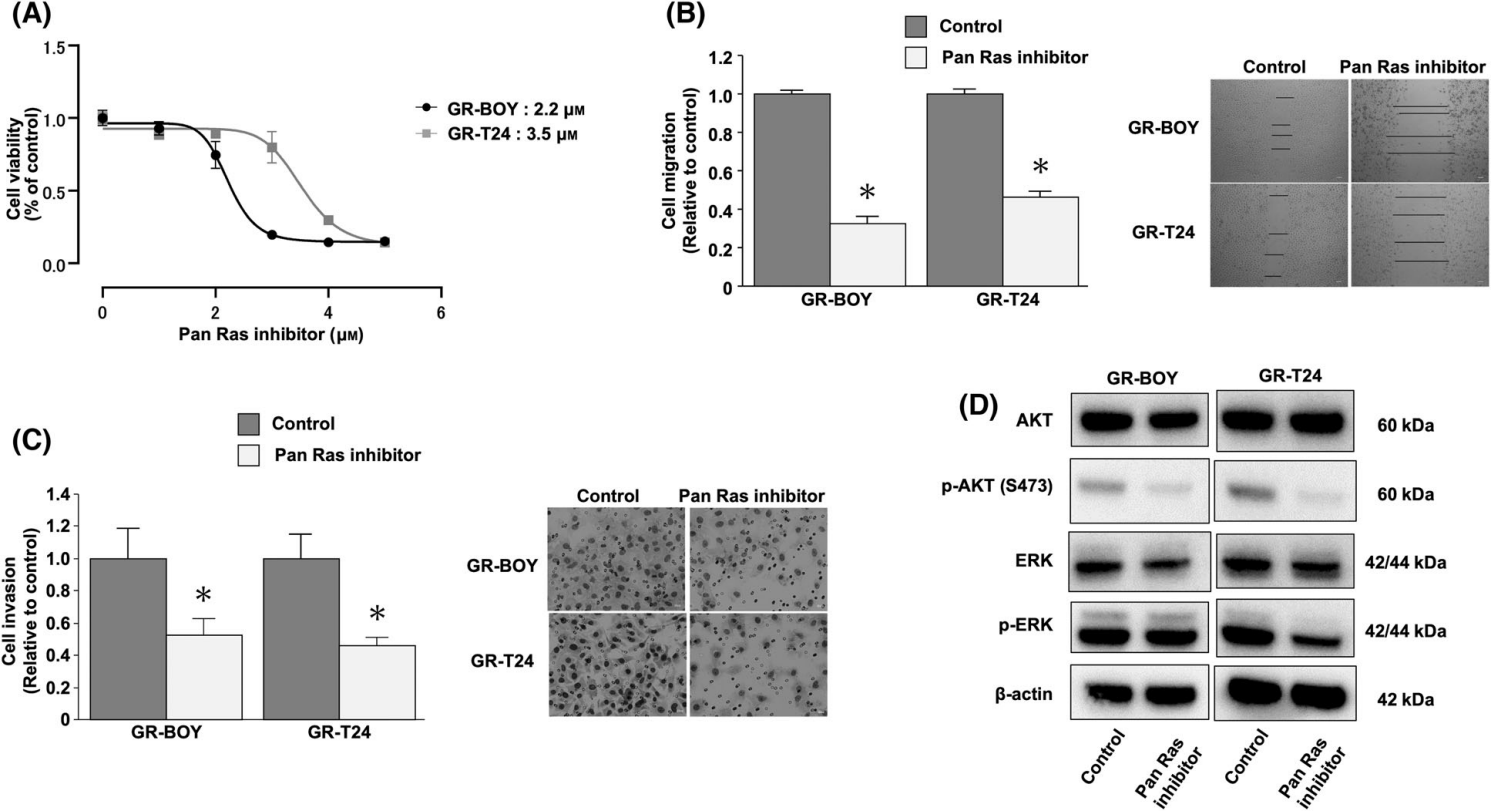
Compound 3144 inhibited cell viability by suppressing RAS‐dependent signaling in GEM‐R BC cells. (A) The IC_50_ values of Compound 3144 in GEM‐R‐BOY and GEM‐R‐T24 cells. *n* = 6. (B) Cell migration (*P < 0.0001. *n* = 12, Mann–Whitney *U* tests) and (C) invasion (*P < 0.05. *n* = 8, Mann–Whitney *U* tests) were determined by wound healing and Matrigel invasion assays, respectively, in GEM‐R‐BOY and GEM‐R‐T24 cells. The length of the scale bar indicates 50 μm (B) and 100 μm (C). The error bars represent SD. (D) GEM‐R‐BOY and GEM‐R‐T24 cells were treated with control (DMSO) or Compound 3144 (5 μm) for 5 h, and RAS‐dependent signaling was evaluated by western blotting. These blots are cropped, and uncropped membranes are shown in Fig. S4. The grouping of blots cropped from different parts of the same gel or from different gels, fields, or exposures was divided with white space.

The effects of Compound 3144 treatment were investigated in GR‐BOY and GR‐T24 cells. Based on the IC_50_ values of cisplatin, Compound 3144 inhibited cell proliferation in GR‐BOY and GR‐T24 cells at relatively low concentrations (GR‐BOY IC_50_: 2.2 μm, GR‐T24 IC_50_: 3.5 μm, Fig. 3A). Moreover, compared with the control, Compound 3144 significantly reduced GR‐BOY and GR‐T24 cell migration (GR‐BOY, control 1.0 ± 0.072, Compound 3144 0.328 ± 0.112, *P* < 0.0001; GR‐T24, control 1.0 ± 0.077, Compound 3144 0.465 ± 0.107, *P* < 0.0001; Mann–Whitney *U* tests; Fig. 3B) and invasion activity (GR‐BOY, control 1.0 ± 0.532, Compound 3144 0.525 ± 0.294, *P* = 0.0460; GR‐T24, control 1.0 ± 0.436, Compound 3144 0.460 ± 0.134, *P* = 0.0239; Mann–Whitney *U* tests; Fig. 3C). Western blot analysis showed that Compound 3144 decreased the phosphorylation of Ser473 on AKT in GR‐BOY and GR‐T24 cells and the phosphorylation of ERK in GR‐T24 cells (Fig. 3D, Fig. S4). By contrast, the phosphorylation of ERK was not decreased by Compound 3144 in GR‐BOY cells.

### Compound 3144 inhibited cell viability by suppressing RAS‐dependent signaling in cisplatin‐resistant BC cells

**Fig. 4:**
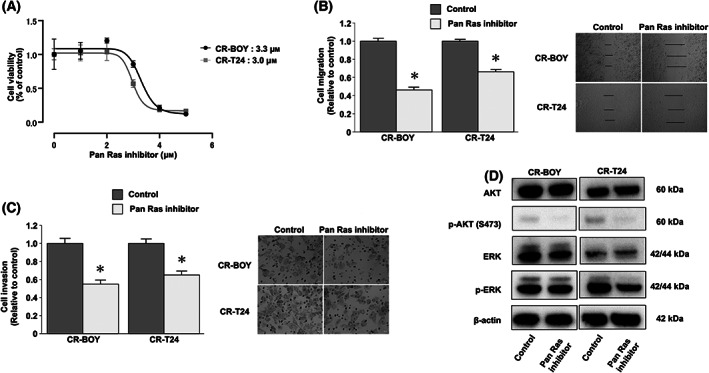
Compound 3144 inhibited cell viability by suppressing RAS‐dependent signaling in CDDP‐R BC cells. (A) The IC_50_ values of Compound 3144 in CDDP‐R‐BOY and CDDP‐R‐T24 cells. *n* = 6. (B) Cell migration (*P < 0.001. *n* = 9, Mann–Whitney *U* tests) and (C) invasion (*P < 0.01. *n* = 8, Mann–Whitney *U* tests) were determined by wound healing and Matrigel invasion assays, respectively, in CDDP‐R‐BOY and CDDP‐R‐T24 cells. The length of the scale bar indicates 50 μm (B) and 100 μm (C). The error bars represent SD. (D) CDDP‐R‐BOY and CDDP‐R‐T24 cells were treated with control (DMSO) or Compound 3144 (5 μm) for 1 h, and RAS‐dependent signaling was evaluated by western blotting. These blots are cropped, and uncropped membranes are shown in Fig. S4. The grouping of blots cropped from different parts of the same gel or from different gels, fields, or exposures was divided with white space.

Next, the effects of Compound 3144 treatment were investigated in CR‐BOY and CR‐T24 cells. Based on the IC_50_ values of cisplatin, Compound 3144 inhibited cell proliferation in CR‐BOY and CR‐T24 cells at relatively low concentrations (CR‐BOY IC_50_: 3.3 μm, CR‐T24 IC_50_: 3.0 μm, Fig. 4A). Moreover, compared with the control, Compound 3144 significantly reduced CR‐BOY and CR‐T24 cell migration (CR‐BOY, control 1.0 ± 0.103, Compound 3144 0.465 ± 0.076, *P* = 0.0003; CR‐T24, control 1.0 ± 0.054, Compound 3144 0.665 ± 0.075, *P* = 0.0003; Mann–Whitney *U* tests; Fig. 4B) and invasion activity (CR‐BOY, control 1.0 ± 0.167, Compound 3144 0.548 ± 0.130, *P* = 0.001; CR‐T24, control 1.0 ± 0.140, Compound 3144 0.650 ± 0.127, *P* = 0.0011; Mann–Whitney *U* tests; Fig. 4C). Western blot analysis showed that Compound 3144 decreased the phosphorylation of Ser473 on AKT and the phosphorylation of ERK in CR‐BOY and CR‐T24 cells (Fig. 4D, Fig. S4).

### Compound 3144 inhibited cell viability by suppressing RAS‐dependent signaling in parental BC cells

Finally, the effects of Compound 3144 treatment were investigated in parental BOY and T24 cells. Based on the IC_50_ values of cisplatin, Compound 3144 inhibited cell proliferation in BOY and T24 cells at concentrations similar to those in gemcitabine‐ and cisplatin‐resistant cells (BOY IC_50_: 2.9 μm, T24 IC_50_: 3.6 μm, Fig. S2A). Furthermore, compared with the control, Compound 3144 significantly reduced BOY and T24 cell migration (BOY, control 1.0 ± 0.094, Compound 3144 0.839 ± 0.101, *P* = 0.0062; T24, control 1.0 ± 0.120, Compound 0.765 ± 0.056, *P* = 0.0007; Mann–Whitney *U* tests; Fig. S2B) and invasion activity (BOY, control 1.0 ± 0.200, Compound 3144 0.431 ± 0.138, *P* = 0.0008; T24, control 1.0 ± 0.110, Compound 3144 0.407 ± 0.083, *P* = 0.0008; Mann–Whitney *U* tests; Fig. S2C). Western blot analysis showed that Compound 3144 decreased the phosphorylation of Ser473 on AKT and the phosphorylation of ERK in parental BOY and T24 cells (Figs S2D and S5).

### Compound 3144 inhibited spheroid formation in GEM‐R, CDDP‐R, and parental BC cells

**Fig. 5:**
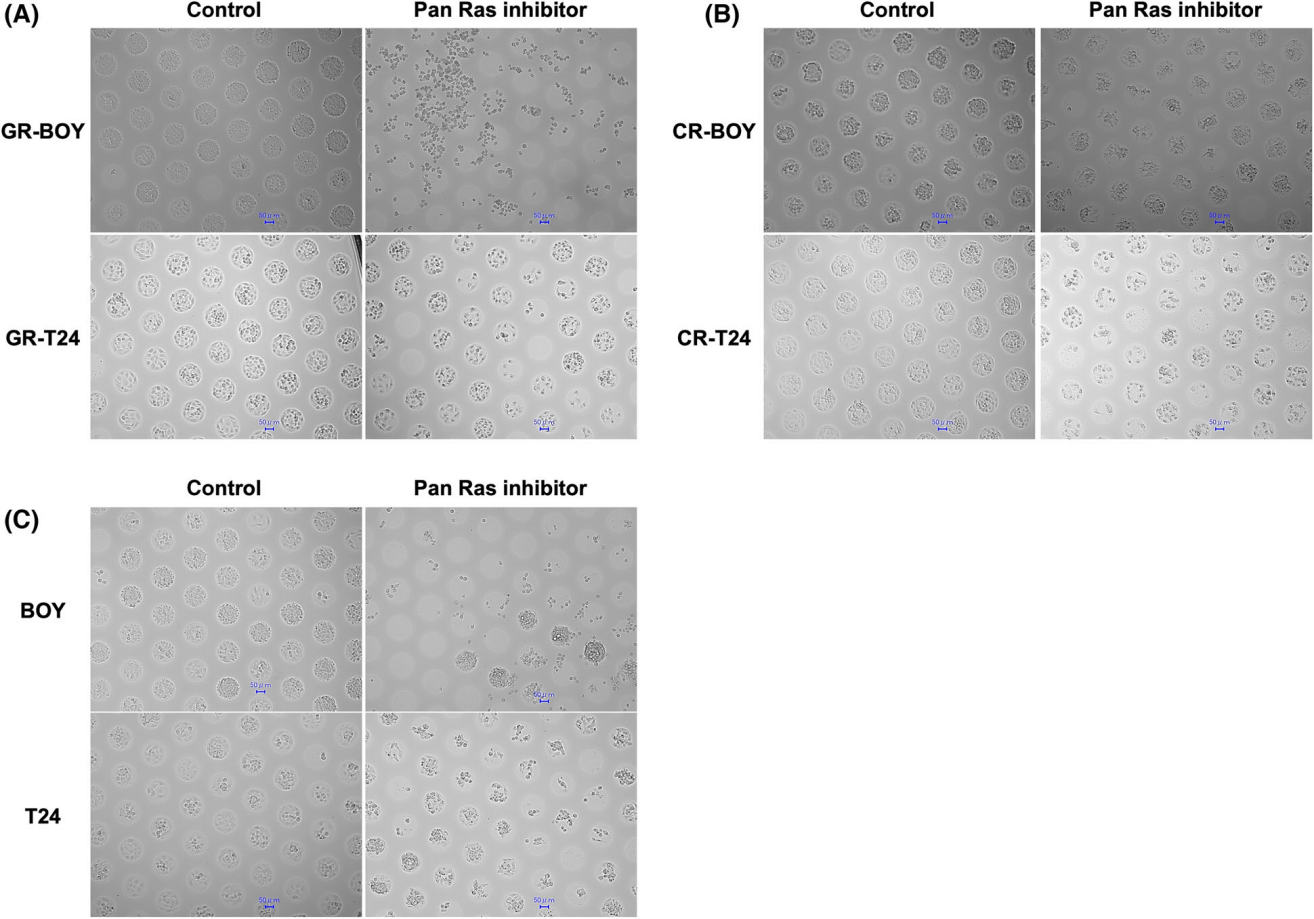
Compound 3144 inhibited spheroid formation in GEM‐R, CDDP‐R, and parental BC cells. GEM‐R‐BOY/T24 (A), CDDP‐R‐BOY/T24 (B), and parental BOY/T24 cells (C) were treated with control (DMSO) or Compound 3144 (5 μm) for 48 h, and Spheroid formations were evaluated respectively. The length of the scale bars indicate 50 μm.

Because cancer stem cells (CSCs) are thought to be responsible for chemoresistance, we investigated the effect of Compound 3144 against tumor spheroid formation ability in both drug‐resistant cells and their parental cells. Compound 3144 obviously inhibited spheroid formation in GEM‐R‐BOY/T24 (Fig. 5A), CDDP‐R‐BOY/T24 (Fig. 5B), and parental BOY/T24 cells (Fig. 5C).

### Compound 3144 inhibited signaling

We next performed RNAseq of the parental, gemcitabine‐resistant, and cisplatin‐resistant BC cell lines after treatment with Compound 3144. We identified 106 genes that were commonly downregulated by Compound 3144, which was less than half that in the control for all 6 cell lines; the expression of 22 genes was completely suppressed by Compound 3144 (Table 1). We also performed Gene Ontology biological processes pathway analyses with 106 genes, and pathways associated with the cell cycle were particularly enriched (Table 2).

**Table 1:** Downregulated genes in BOY/T24, CR‐BOY/T24, and GR‐BOY/T24 by Pan‐RAS inhibitor.

GeneID	GeneSymbol	Description
5152	*PDE9A*	Phosphodiesterase 9A
10811	*NOXA1*	NADPH oxidase activator 1
23254	*KAZN*	Kazrin, periplakin interacting protein
23409	*SIRT4*	Sirtuin 4
23416	*KCNH3*	Potassium voltage‐gated channel subfamily H member 3
51134	*CEP83*	Centrosomal protein 83
54932	*EXD3*	Exonuclease 3′–5′ domain containing 3
57571	*CARNS1*	Carnosine synthase 1
79802	*HHIPL2*	HHIP like 2
85007	*PHYKPL*	5‐phosphohydroxy‐l‐lysine phospho‐lyase
121512	*FGD4*	FYVE, RhoGEF and PH domain containing 4
136227	*COL26A1*	Collagen type XXVI alpha 1 chain
145482	*PTGR2*	Prostaglandin reductase 2
388581	*C1QTNF12*	C1q and TNF related 12
554235	*ASPDH*	Aspartate dehydrogenase domain containing
100129520	*TEX13C*	TEX13 family member C
100507433	*ZNF571‐AS1*	ZNF571 antisense RNA 1
101928570	*LOC101928570*	Uncharacterized LOC101928570
101928931	*SEMA3B‐AS1*	SEMA3B antisense RNA 1 (head to head)
101929823	*LOC101929823*	Uncharacterized LOC101929823
105372431	*LOC105372431*	Uncharacterized LOC105372431
105374366	*LINC02482*	Long intergenic non‐protein coding RNA 2482

**Table 2:** Gene Ontology Biological Processes downregulated in BOY/T24, CR‐BOY/T24, and GR‐BOY/T24 by Pan‐RAS inhibitor.

Description	Number of genes	*P*‐value	Genes
Cell cycle	13	0.00000001	*DMC1, PKMYT1, SKA3, UBE2C, NCAPG2, CENPX, FOXM1, VRK1, AURKA, KLHL21, SMC2, ZWINT, NUDC*
Cell division	10	0.00000019	*SKA3, UBE2C, NCAPG2, CENPX, VRK1, AURKA, KLHL21, SMC2, ZWINT, NUDC*
Spindle organization	3	0.00040989	*RANBP1, AURKA, NTMT1*
Mitotic cell cycle	5	0.00049491	*PKMYT1, SKA3, TUBB6, AURKA, GEM*
Signal transduction	12	0.00061727	*PDE9A, ITGAL, C1QTNF12, TFRC, LPXN, KSR1, EDA, RANBP1, LRRC59, ASIP, GEM, TXNRD1*
Chromosome organization	3	0.00097515	*RAD54L, GEM, SMC2*
Mitotic spindle organization	4	0.00264234	*AURKA, CENPN, ZWINT, NUDC*
Meiotic cell cycle	4	0.00273986	*RAD54L, DMC1, PKMYT1, AURKA*
Cellular response to DNA damage stimulus	6	0.00786887	*RAD54L, ZNF385A, CENPX, FOXM1, SIRT4, IMMP2L*
Regulation of cell growth	3	0.00999275	*KAZALD1, TFRC, FOXM1*

### RAS status based on the BLCA cohort of TCGA

The analysis with TCGA database in BC indicated that HRAS and KRAS mutations were observed in nearly 5%, whereas only 1% was mutated in NRAS. On the other hand, mRNA high expression or DNA amplification was more frequently observed than mutations in each RAS (Fig. S3). In total, one fourth of BCs had RAS mutation, its mRNA high expression or its DNA amplification.

## Discussion

The mechanisms of acquisition of cisplatin resistance can be classified into 4 categories [16]. The first category is pre‐target resistance, which involves steps preceding the binding of cisplatin to DNA, perhaps owing to lowered cisplatin uptake into cells. The second category is on‐target resistance related to inadequate DNA/cisplatin adducts. The third category is post‐target resistance owing to the lethal signaling pathways elicited by cisplatin‐mediated DNA damage. The last category is off‐target resistance, in which a nontarget signaling pathway is triggered by cisplatin [16]. Several mechanisms have also been identified for the mechanisms of acquisition of gemcitabine resistance [17]. The first mechanism of gemcitabine resistance is the dysregulation of proteins associated with gemcitabine metabolism through human equilibrative nucleoside transporter 1, downregulation of the rate‐limiting enzyme deoxycytidine kinase (dCK), upregulation of ribonucleotide reductase catalytic subunit M (RRM) 1/RRM2, and Hu antigen R, an RNA‐binding protein that post‐transcriptionally regulates dCK. The second mechanism is high expression of drug efflux pumps, including ABC transporter family proteins, to protect CSCs from chemotherapeutic agents. The epithelial‐mesenchymal transition has also been reported to be associated with CSCs. Another mechanism involves multiple genetic and epigenetic abnormalities, such as mutations in nuclear factor‐κB, AKT, mitogen‐activated protein kinase, and hypoxia‐inducible factor‐1α pathways, which lead to inactivation of apoptosis. However, few reports have described concurrent GC resistance. Pan et al. [18] showed that the long noncoding RNA *UCA1* promotes cisplatin/gemcitabine resistance through CREB‐modulating *miR‐196a‐5p* in BC cells. In our study, RNAseq showed that there were common alterations in gene expression related to acquisition of drug resistance to GC, even in different cell lines; the results also suggested that there may be different mechanisms mediating GC resistance. Although there was no apparent cross‐resistance between gemcitabine‐ and cisplatin‐resistant cells because the functional mechanisms of these drugs differ, to the best of our knowledge, our finding that there was no cross‐resistance between these resistant cell lines was the first report of this result obtained using drug‐resistant cell lines.

Recently, the KRAS G12C inhibitor sotorasib was approved as the world's first drug targeting KRAS, showing efficacy against unresectable advanced or recurrent non‐small cell lung cancer with KRAS G12C mutation‐positive disease that progressed after cancer chemotherapy [19]. However, KRAS and NRAS mutations are less frequently observed in BC, whereas HRAS mutations occur in 4% of BC cases [11]. Actually, TCGA database in BC indicated that HRAS and KRAS mutations were observed in nearly 5%, whereas only 1% was mutated in NRAS. On the other hand, mRNA high expression or DNA amplification was more frequently observed than mutations in each RAS (Fig. S3). In total, one fourth of BCs had RAS mutation, mRNA high expression or DNA amplification. Therefore, a pan‐RAS inhibitor, such as Compound 3144, may be beneficial for the treatment of BC, as shown in this study. However, Compound 3144 showed toxicity and off‐target activity, despite its excellent antitumor efficacy, which it exerts via binding with wild‐type KRAS, NRAS, and HRAS [14]. In addition, pan‐RAS inhibitors are unlikely to be tolerated because RAS function is critical in normal cells [20]. Recently, enfortumab vedotin, a nectin‐4‐directed antibody and microtubule inhibitor conjugate, was approved for the treatment of urothelial cancer in patients who had previously received a platinum‐containing chemotherapy and a PD‐1 or PD‐L1 inhibitor [21, 22]. Enfortumab vedotin is an antibody‐drug conjugate (ADC) that combines the targeting capabilities of monoclonal antibodies with the cancer‐killing ability of cytotoxic drugs [22]. Because ADCs can be designed to distinguish between healthy and diseased tissue [23], these compounds may be an effective method for delivery of pan‐RAS inhibitors to tumors for applications in the clinical setting.

The novel pan‐RAS inhibitor Compound 3144, which was designed to target multiple sites on RAS proteins, showed sufficient affinity and selectivity for pharmacological inhibition of RAS signaling, e.g., the RAS/PI3K/AKT and RAS/RAF/MEK/ERK cascades [14]. In our study, phosphorylation of AKT was sufficiently suppressed by Compound 3144 in parental and drug‐resistant BC cells, whereas phosphorylation of ERK was not suppressed, particularly in BOY cells. These findings may be related to the specific characteristics of the cell lines. For example, T24 cells harbor the HRASG12V mutation (a substitution of glycine by valine at codon 12 of HRAS), and BOY cells contain wild‐type HRAS [13]. RNAseq analysis of cells treated with Compound 3144 also identified several genes that showed complete downregulation, and several pathways, particularly cell cycle‐related pathways, were associated with Compound 3144 treatment. Because RNAseq analysis identified different genes and pathways for known mechanisms associated with GC resistance, Compound 3144 could inhibit GR and CR BC cells. Notably, Compound 3144 inhibited not only cell proliferation but also cell migration and invasion in this study. Therefore, further studies are needed to elucidate the functional roles of Compound 3144 *in vitro* and *in vivo*.

## Conclusions

In this study, we found that cross‐resistance did not occur between gemcitabine‐ and cisplatin‐resistant BC cells. Moreover, Compound 3144 showed antitumor effects in both gemcitabine‐ and cisplatin‐resistant BC cells. Our findings provided important insights into the mechanisms of GC resistance in BC and may facilitate the development of novel therapeutic strategies to treat progressive BC.

## Conflict of interest

The authors declare no conflict of interest.

## Author contributions

HY designed the study; SY and MT analyzed data; and HY finalized the manuscript; MT, SO, and S Iizasa performed experiments and collected and analyzed data; HY, TS, YO, S Inoguchi, RM, YY, MN, ST, AT, and HE secured research funding and drafted the article. All authors were involved in writing the manuscript and reviewed and approved the final version.

## Supporting information


**Fig. S1.** Heat map from RNAseq analyses of parental and drug‐resistant cells. Clustering analysis of mRNA expression levels. High mRNA expression levels are indicated in red, and low levels are indicated in blue.

**Fig. S2.** Compound 3144 inhibited cell viability by suppressing RAS‐dependent signaling in parental BC cells. (A) The IC_50_ values of Compound 3144 for parental BOY and T24 cells. n = 6. (B) Cell migration (*P < 0.01. n = 9, Mann–Whitney U tests) and (C) invasion (*P < 0.001. n = 8, Mann–Whitney U tests) were determined by wound healing and Matrigel invasion assays, respectively, in parental BOY and T24 cells. The length of the scale bar indicates 50 μm (B) and 100 μm (C). The error bars represent SD. (D) Parental BOY and T24 cells were treated with control (DMSO) or Compound 3144 (5 μM) for 1 h, and RAS‐dependent signaling was evaluated by western blotting. These blots are cropped and uncropped membranes are shown in Fig. S5. The grouping of blots cropped from different parts of the same gel or from different gels, fields, or exposures was divided with white space.

**Fig. S3.** RAS status based on the BLCA cohort of TCGA. Genetic alteration of RAS genes in BC samples.

**Fig. S4.** The full‐length blots/gels of Figs 3D and 4D.

**Fig. S5.** The full‐length blots/gels of Fig. S2D.

## Data Availability

The data that supports the findings of this study are available in the Supplementary Material of this article.
